# Sensory nerve action potentials and sensory perception in women with arthritis of the hand

**DOI:** 10.1186/1743-0003-9-27

**Published:** 2012-05-10

**Authors:** Kristina M Calder, Alison Martin, Jessica Lydiate, Joy C MacDermid, Victoria Galea, Norma J MacIntyre

**Affiliations:** 1 School of Rehabilitation Science, McMaster University, IAHS-Room 403, 1400 Main Street West, Hamilton, ON L8S 1C7, Canada

**Keywords:** Osteoarthritis, Rheumatoid arthritis, Nerve conduction study, Sensory perception, Sensory loss

## Abstract

**Background:**

Arthritis of the hand can limit a person’s ability to perform daily activities. Whether or not sensory deficits contribute to the disability in this population remains unknown. The primary purpose of this study was to determine if women with osteoarthritis (OA) or rheumatoid arthritis (RA) of the hand have sensory impairments.

**Methods:**

Sensory function in the dominant hand of women with hand OA or RA and healthy women was evaluated by measuring sensory nerve action potentials (SNAPs) from the median, ulnar and radial nerves, sensory mapping (SM), and vibratory and current perception thresholds (VPT and CPT, respectively) of the second and fifth digits.

**Results:**

All SNAP amplitudes were significantly lower for the hand OA and hand RA groups compared with the healthy group (*p* < 0.05). No group differences were found for SNAP conduction velocities, SM, VPT, and CPT.

**Discussion:**

We propose, based on these findings, that women with hand OA or RA may have axonal loss of sensory fibers in the median, ulnar and radial nerves. Less apparent were losses in conduction speed or sensory perception.

## Background

Osteoarthritis (OA) and rheumatoid arthritis (RA) of the hand significantly limit a person’s ability to perform daily activities [1-5]. It has been hypothesized that limitation in activities is primarily related to pain; however, sensory factors contributing to the disability of these diseases remain unknown [2,4]. Identifying sensory abnormalities in other upper extremity conditions has resulted in treatment approaches that incorporate motor control or sensory retraining in rehabilitation programs. For this reason, a better understanding of the sensory deficits present in OA and RA affecting the hand might contribute to the design of future rehabilitation programs.

Neurophysiological investigations have been performed on persons with RA of the hand where mild sensory changes resulting from peripheral neuropathy of the forearm have been reported [6-9]. Often it is difficult to diagnosis upper limb neuropathy in RA because of disuse atrophy of the muscles controlling hand and digit movements. However, persons with RA are commonly diagnosed with carpal tunnel syndrome which contributes to both sensory and motor changes in the median distribution of the hand. The prevalence of a peripheral neuropathy has been reported to be 23–69% in persons with RA [10]. In most cases, diagnosing a neuropathy in RA is of secondary importance as physicians tend to focus more attention on controlling joint inflammation [11]. Thus undetected local or more generalized neuropathy may contribute to functional difficulties in people with RA.

Relatively few neurophysiological investigations have been reported in persons with OA affecting the hand [12,13]. Sensory perception abnormalities have been observed in persons with lower limb OA [14-17], and these have been proposed to contribute to functional disability. It is reasonable to suspect that similar abnormalities in the hand could contribute to disability in persons with OA of the hand.

Muscle function has been well studied in persons with hand OA or RA. Decreased hand strength is a commonly reported impairment, specifically grip and pinch strength [18-20]. Furthermore, persons with OA or RA of the hand perform less well during hand dexterity tasks, and have lower self-rated hand function when compared to healthy participants [21-24]. One theory proposes that changes in muscle loading around the joints due to muscle atrophy and subsequent joint deformity contribute to deficits in strength and dexterity in persons with RA [25]. Although the majority of the research demonstrating changes to muscle loading and activity have been in persons with OA or RA of the knee [26-31], there is evidence of reduced muscle activation levels in the first dorsal interossoeus muscle during maximal grip contractions in persons with RA of the hand [32]. In contrast, no alterations in muscle activity were detected in women with hand OA performing a hand dexterity task [13]. Since sensory feedback contributes to motor performance, it is important to consider alterations in sensory perception as possible contributors to disability of the hand.

The primary purpose of this study was to determine if women with OA or RA of the hand demonstrate sensory impairments of the hand compared to women without hand problems. The status of the sensory system was measured using both neurophysiological and sensory perception tests. The secondary purpose of this study was to determine if the status of the sensory system was related to self-reported disability, strength, and dexterity of the hand.

## Methods

### Participants

Participants were community-dwelling women between the ages of 50 and 65 years without hand problems and those who met the American College of Rheumatology clinical criteria for OA or RA of the dominant hand [33]. This age group was chosen based on epidemiological evidence that hand OA has its highest prevalence in women over the age of 50 [1-3], and significant age-related muscle fiber atrophy and motor unit loss start to occur in the distal muscles in the 60- to 79-year age group [34]. Participants were recruited through advertisements on the website of a local branch of the Arthritis Society, our institutional website, posters in community facilities and local rheumatology clinics, and by word of mouth. People who expressed interest in the study were screened for eligibility by email or over the telephone. Exclusion criteria were: type II diabetes requiring insulin therapy, fracture or other injuries of the dominant hand, compression neuropathy, finger amputation on the dominant hand, joint replacement in the fingers or the wrist of the dominant hand, and presence of other rheumatic diseases which may have altered hand function for reasons unrelated to OA or RA. This study was approved by our institutional research ethics review board for studies involving human participants, and informed consent was obtained from all participants.

### Design

This was a cross-sectional group comparison study. All participants had the dominant hand tested for sensory and motor action potentials, sensory perception, dexterity and strength. A physical therapist examined the dominant hand and wrist of each participant and recorded pain, swelling, deformity, and nodes. The participants with arthritis also completed a self-report questionnaire on their hand disability. The single test session lasted 2 to 3 hours. Neurophysiological and sensory perception testing order was randomly assigned by drawing numbers prior to each session.

### Neurophysiological tests

The temperature in the laboratory was maintained at a constant temperature of 23 ± 2 degrees Celsius during the neurophysiological tests. This is the room temperature suggested so that the skin temperature is at least 32 degrees Celsius.

### Sensory nerve action potentials

**Figure 1:**
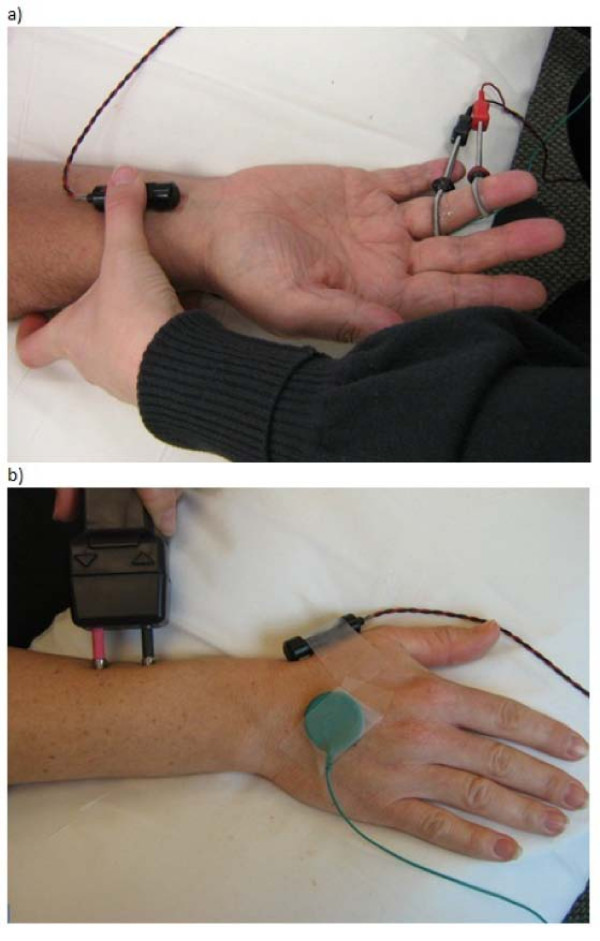
**Experimental set-up for recording sensory nerve action potentials (SNAPs).** ( **a**) For the median and ulnar nerves, ring electrodes are placed over the 4th digit and the nerve is stimulated 13 cm proximal to the closest recording electrode. This image shows the stimulating site for the median nerve. ( **b**) For the radial nerve, the bipolar recording electrode is placed distal to the ‘snuff box’, and the stimulating probe is placed 10 cm proximal to the closest recording electrode. The reference electrode is placed on the back of the hand for all tests.

The sensory nerve action potentials (SNAPs) of the median, ulnar and radial nerves were recorded using the XLTEK NeuroMax EMG system (Natus Medical Incorporated, San Carlos, CA USA). This test involves stimulation of the nerves and recording an action potential antidromically using a bipolar electrode configuration that has been shown to give adequate SNAP amplitude, latency and conduction velocity readings [35-37]. Antidromic stimulation of the nerve results in larger SNAP amplitudes versus orthodromic stimulation, particularly in the distal median and ulnar nerves [38]. Given that large myelinated A_α_ or A_β_ fibers tend to have lower thresholds when artificially stimulated, nerve conduction velocities are primarily a reflection of conductivity of these nerve fibers. For all testing, the participant was seated comfortably in a chair with her hand and forearm resting on a pillow in her lap. Prior to the placement of electrodes, the application sites were thoroughly cleaned with rubbing alcohol. A reference electrode was placed on the posterior aspect of the hand. For the median and ulnar recordings, two ring electrodes were secured around the proximal and distal phalanges of digit four. The stimulator was placed over the nerve of interest 13 cm proximal to the most proximal recording electrode (Figure 1A). For the radial nerve recording, a bipolar surface electrode was positioned distal to the anatomical “snuff box” and proximal to the metacarpophalangeal joint of the first digit. The stimulator was positioned over the radial nerve 10 cm proximal to the most proximal recording electrode (Figure 1B).

For all testing, the duration of the stimulus was set to 200 ms and the stimulus current was initially set to zero. Following an elicited response, the primary investigator visually inspected the waveform to ensure it had a distinct onset and was biphasic in shape. The stimulus was increased incrementally until the SNAP amplitude reached its maximum point. Supramaximal stimulation was set to 120% of this intensity, and ten readings of each nerve were obtained and averaged [37]. The amplitude of the SNAP was the vertical distance from baseline to the negative peak; latency was the time from peak of stimulus artifact to the negative peak of the waveform; and conduction velocity was the distance between stimulus and recording electrodes divided by latency [36].

### Neuromuscular recording

Compound muscle action potentials (CMAPs) were also measured as part of the nerve conduction testing to further clarify the overall state of the participant’s peripheral nerve physiology. A CMAP provides information regarding the muscle size as it is the summation of individual motor unit action potentials [39].

An active electrode was positioned over the motor point of the abductor pollicis brevis muscle, with a reference surface electrode positioned over the first metacarpophalangeal joint making a monopolar configuration. The stimulus duration was set at 100 ms, and the stimulus current was initially set to zero and increased incrementally until maximum potential amplitude was achieved. Ten CMAPs were recorded orthodromically at supramaximal stimulation levels (120%; CMAP1). Following stimulation of the median nerve at the wrist (7 cm proximal to the active recording electrode), the stimulator was moved up to the antecubital fossa, and once the supramaximal stimulation level was found, ten more recordings were performed (CMAP2). The distance between the active recording electrode and the stimulation site at the antecubital fossa was used in the equation to calculate conduction velocity along the forearm. During offline analysis, the average latencies (measured from the peak of the stimulus artifact to the onset of the waveform) and amplitudes (negative peak to positive peak) were calculated for the CMAP1 and CMAP2 sites. Average conduction velocity was calculated by measuring the distance between the two stimulation sites and dividing by the difference between the proximal and distal latencies. This test has a normal upper limit of 4.4 ms for latency, a normal lower limit of 4 mV for amplitude, and a normal lower limit for conduction velocity of 49 m/s [36].

### Sensory perception testing

Sensory perception was evaluated using three different quantitative sensory tests.

### Sensory mapping test

**Figure 2:**
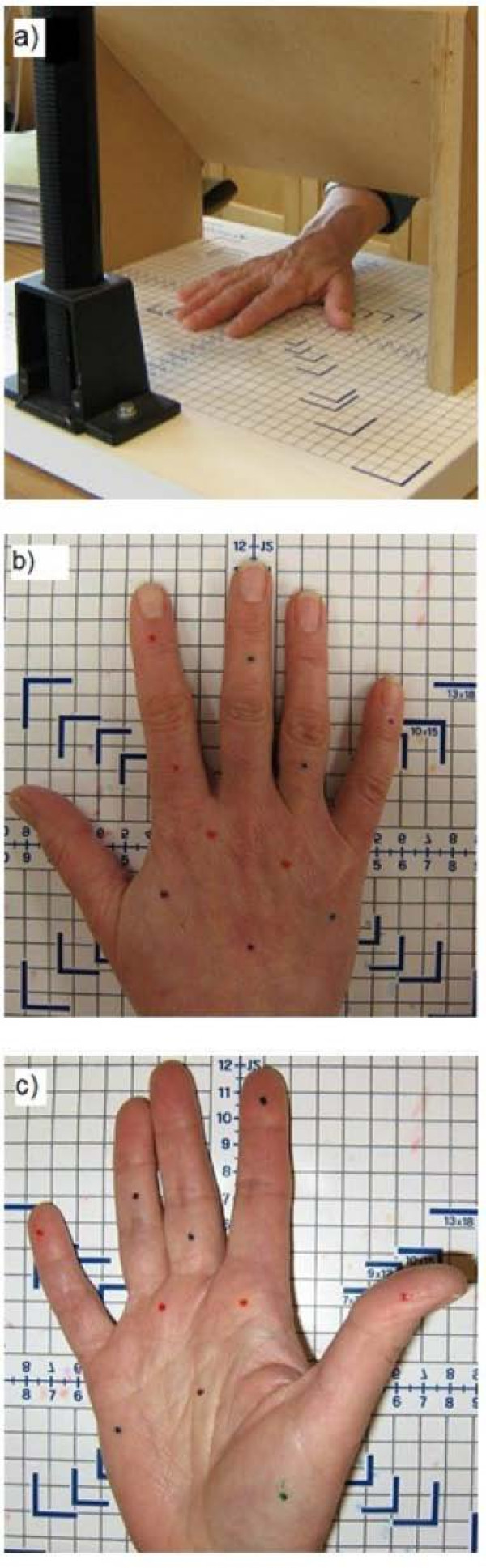
**Experimental set-up for the sensory mapping (SM) task.** ( **a**) The hand is placed under a shield to block the participant’s view. Predetermined marks are placed on the ( **b**) dorsal hand and ( **c**) palmar hand.

The ability to localize a sensory stimulus on the body surface (locognosia) was tested using a sensory mapping (SM) technique. SM was used to quantify the ability of the participants to locate where they were touched on the hand using a method similar to that used for clinical assessment of patients with nerve injury and shown by others to provide reliable scores [40]. In our testing protocol, the participants were seated with their arm resting on a pillow placed on their lap. The elbow was flexed to 90° and the forearm was pronated in order to position the hand under a screen which prevented visual feedback (Figure 2a). An aerial view photo was taken of the dorsal hand. Immediately following the photo, a predetermined pattern of 10 dots was drawn onto the participant’s hand with washable marker, and another photo was taken (Figure 2b). The forearm was then supinated and the same procedure was repeated on the palmar hand (Figure 2c). The photos were printed off and those of the unmarked hand were placed beside the participants. With the hand still blocked from view, the examiner re-stimulated each of the 10 dots with a fine tipped object. The participants used their non-dominant hand to mark on the photo the location where they perceived the stimulus. Following testing, transparencies were made of the photos depicting the pattern of dots placed by the investigator. These transparencies were laid over the photos depicting the pattern of dots as indicated by the participants. The distance between the dots representing each actual and perceived point of stimulus were measured (mm) and the average for all 10 points on the dorsal hand and all 10 points on the palmar hand were recorded.

### Vibratory Perception Threshold

Vibratory perception threshold (VPT) was tested on the distal pulp of the 2^nd^ and 5^th^ digits using a Jtech vibrometer (Vibrometer PCV50; Jtech, Salt Lake City, UT) that has a 2 mm diameter aperture surrounding a 1 mm diameter vibrating post. The post vibrates at a constant frequency of 50 Hz while the amplitude of the vibration is modulated by a displacement-controlled actuator. The VPT has been shown to be a simple and reliable technique for assessing sensory loss in persons with OA of the hip or knee or with neuropathies [16,17,41,42].

The participants were seated comfortably with their forearm pronated and supported on a table, the elbow flexed to 90° and the distal pulp of the finger resting lightly on the vibrating post. The participants held the interface controller in their non-dominant hand and depressed the button on it when they perceived a vibration.

Prior to testing, a sample suprathreshold stimulus was given to each participant. During testing, each stimulus lasted for 1 second followed by a 3 second interval of no stimulus. The participants had to depress the button within 2 seconds of stimulus onset for the amplitude to be recorded as perceived. The staircase method of threshold determination was programmed into the vibrometer and was used to calculate the minimum vibration amplitude (μm) that could be detected by the participants. In total, three trials were completed on each digit, and the average of trials 2 and 3 was used as the outcome [41]. The computer was positioned out of the participant’s view and the participants were informed when each trial was completed.

### Current Perception Threshold

The current perception thershold (CPT) was measured on of digit 2 and digit 5 using a Neurometer® (Neurotron Inc., Baltimore, Maryland, USA, 2002–3) at 5, 250 and 2000 Hz. The CPT Neurometer® is a device for evaluating and quantifying impairment of sensory nerve fibers (large myelinated A_β_-fibers, thin myelinated A_δ_-fibers, and unmyelinated C-fibers) [43].

The participants were seated with the elbow flexed to 90° and the pronated forearm supported on a table in front of the CPT device. The participant-operated input device was positioned in front of their nondominant hand which was also resting comfortably on the table. The constant current outputs were delivered to the digit skin surface at the tip via a pair of 1 cm diameter gold electrodes separated by 1.7 cm. Prior to electrode placement, the skin was cleaned with rubbing alcohol and the disk electrodes were filled with a thin layer of conductive gel and taped onto the digit tip being tested (digit 2 or digit 5). For each of the sine wave frequencies the intensity was increased (0–10 mA) until the participants experienced a painless sensation at or near the electrode. Next, the participants were presented with 7 to 10 randomly generated sets of stimuli above and below their level of perception and asked to choose which of the two stimuli felt stronger using an automated forced choice protocol. This intensity alignment procedure is conducted to approximate the sensory threshold with a ±50 μAmpere (μA) range out of a total range of 0 to 9.99 mAmperes. The device automatically adjusts the level of stimulation based on the participant’s response. A CPT value (μA) based on the minimal current perceived was calculated once a sufficient number of correct consecutive responses had been obtained.

### Self-report disability and performance tests

Participants with arthritis of the hand completed the Hand and Finger Function scale of the Arthritis Impact Measurement Scales 2 (AIMS-2). This questionnaire has been used extensively in evaluating disability and health status in persons with OA and RA [44]. The Hand and Finger Function scale has 5 items that are scored from 5 (most disability) to 1 (able to perform all activities all days of the week). A total score was calculated by summing the item scores, subtracting 5 from the total and dividing by 2. The resulting values could range from 0–10 with a higher score representing greater disability in hand activities.

Muscle strength and dexterity were evaluated on the NK Hand Assessment System (NK Biotechnical Corporation, Minneapolis, MN, USA). Protocols with established reliability were used to assess maximum tripod pinch strength (kg) and maximum grip strength (kg) and the average of triplicate trials was recorded [45,46]. Dexterity was evaluated by determining the time (s) taken to manipulate the small objects and the large objects on the NK dexterity board. Moderate to high reliability and validity have been previously established for these tests [47,48]. The average time taken to complete three trials was used as the criterion measure for each test (small and large objects).

### Statistics

Statistical analysis was performed using SPSS software (SPSS, Chicago, IL, V.18). Data were tested for normality using the Shapiro-Wilk test. One-way analyses of variance (ANOVAs) were used to compare the demographics (age, height, and weight), self-report disability (AIMS-2 Hand and Finger Function scale), strength measures, dexterity and neuromuscular recordings (CMAP conduction velocity and amplitude) of the women in the healthy, hand OA and hand RA groups. Two-way repeated-measures ANOVAs (group by stimulation site) were used on all sensory measures. The stimulation site levels were: median, ulnar and radial nerve for SNAP amplitude and conduction velocity; palmar and dorsal for SM, and digit 2 and digit 5 for VPT and CPT at each of the three frequencies (5 Hz, 250 Hz, and 2000 Hz). Post hoc analysis was performed using Tukey’s test for pairwise comparisons where appropriate. Cohen’s *d* effect sizes were reported for all analyses due to the small sample size and pilot nature of this study. Alpha level was set at 0.05 for all tests.

Pearson correlation coefficients were used to characterize the associations between selected neurophysiological and sensory perception tests and disability and performance tests for each of the groups.

## Results

### Participants

Nine healthy comparison women, eleven women with hand OA, and seven women with hand RA participated in the study. Table 1 presents the demographic characteristics and observations on clinical examination of the dominant hands for each group. The groups did not differ with respect to age, height, and weight. Three women with hand OA and six women with hand RA had nodes. Only women with hand RA had swollen digits (n = 4). Disease duration was 7.0 ± 4.7 years for the group with hand OA and 14.0 ± 9.9 years for the group with hand RA.

**Table 1:** Mean values and SDs for the demographic characteristics for the women in the healthy, hand OA and hand RA groups and frequency (locations) of nodes and swelling in the digits of the hands in the OA and RA groups

**Characteristics**	**Healthy group** **(n = 9)**	**Hand OA group** **(n = 11)**	**Hand RA group** **(n = 7)**
Age (yr)	56.67 ± 5.50	57.00 ± 5.33	60.43 ± 5.00
Height (cm)	164.98 ± 6.40	162.89 ± 5.02	165.09 ± 4.63
Weight (kg)	65.33 ± 8.72	73.66 ± 14.24	75.58 ± 14.90
**Clinical exam**		**n(location)**	**n(location)**
**Nodes**			
Digit I			1(MCP)
Digit II		3(DIP)	3(DIP); 1(PIP)
Digit III		2(DIP)	2(DIP); 1(PIP)
Digit IV		1(DIP)	1(DIP); 1(PIP)
Digit V			2(DIP);1(PIP);1(MCP)
**Swelling**			
Digit I			2(MCP)
Digit II			4(MCP)
Digit III			1(MCP)
Digit IV			
Digit V			

*OA* osteoarthritis; *RA* rheumatoid arthritis; *MCP* metacarpophalangeal joints; *DIP* distal interphalangeal joints; *PIP* proximal interphalangeal joints.

*Significant difference at the 0.05 level.

### Neurophysiological evaluation

**Figure 3:**
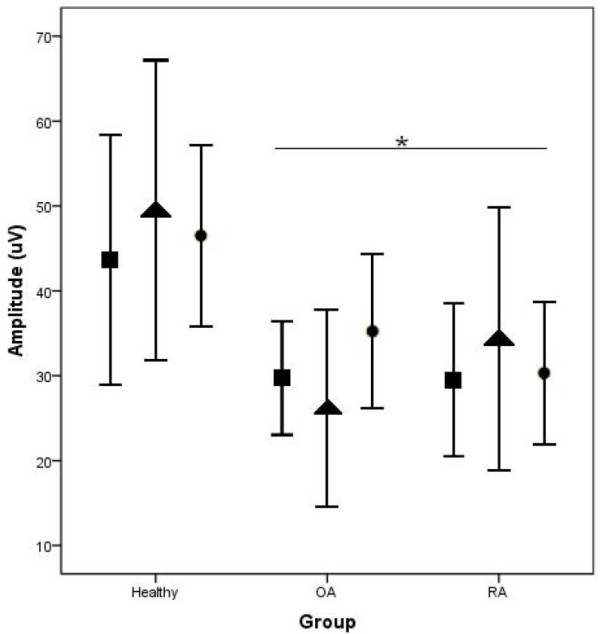
**Mean (SE) values for the sensory nerve action potential (SNAP) amplitudes in the median (■), ulnar (▴) and radial (●) nerves for the women in the healthy group (n = 9), hand OA group (n = 11) and hand RA (n = 7) group.** * significantly different from healthy group.

The SNAP data were collected from eight, nine, and seven women in the healthy, hand OA, and hand RA groups, respectively, due to an inability to record a response in one of the nerves in three of the participants. The ANOVA revealed a significant group effect (F(2,21) = 5.10, *p* = 0.016) for the SNAP amplitudes, but no significant site effect (F(1,21) = 0.228, *p* > 0.05) or group-site interaction (F(2,21) = 0.367, *p* > 0.05). Post hoc analysis indicated that the SNAP amplitude in the healthy group was greater than in the hand OA and hand RA groups (Figure 3).

For SNAP conduction velocity, there were only significant site effects (F(2,21) = 12.61, *p* < 0.001), with faster conduction velocity in the radial nerve compared to the median and ulnar nerves (Table 2). The SNAP latency (Table 2) was recorded for descriptive purposes and to compare with normal values.

**Table 2:** Mean values and SDs of sensory nerve action potential (SNAP) conduction velocity and latency for the median, ulnar and radial nerves of the healthy, hand OA and hand RA groups

**Variable**	**Healthy group** **n = 8**	**Hand OA group** **n = 9**	**Hand RA group** **n = 7**
**SNAP Conduction Velocity (m/s)**			
Median	42.7 ± 6.9	42.6 ± 4.3	43.5 ± 6.3
Ulnar	43.0 ± 6.0	41.3 ± 9.1	42.7 ± 8.0
Radial*	47.3 ± 5.1	51.4 ± 4.4	49.7 ± 8.8
**SNAP Latency (ms)**			
Median	3.1 ± 0.5	3.1 ± 0.3	3.1 ± 0.4
Ulnar	3.1 ± 0.5	3.3 ± 0.9	3.1 ± 0.6
Radial	2.2 ± 0.3	2.0 ± 0.2	2.1 ± 0.4

*significantly greater compared to ulnar and median nerves.

### Sensory perception testing

Mean and SD values for the SM, VPT and CPT tests are shown in Table 3. The CPT could not be calculated for all participants due to an insufficient number of correct consecutive responses. Significant site effects were revealed for the SM (F(1,24) = 14.389, *p* = 0.001) and VPT tests (F(1,24) = 4.465, *p* = 0.045), but not for the CPT test. The SM error was lower on the palmar than on the dorsal hand, and VPT was lower at digit 5 than at digit 2. No significant group or interaction effects were noted for either test. The sample size at 2000 Hz was too small to perform statistical analysis.

**Table 3:** Mean values and SDs for sensory perception measures, sensory mapping (SM) mean errors, vibratory perception thresholds (VPT) and current perception thresholds (CPT) of the healthy, hand OA and hand RA groups

**Variable**		**Healthy group**	**Hand OA group**	**Hand RA group**
SM (mm)^*^	***n***	9	11	7
Mean Error: Dorsal		126.3 ± 39.8	112.7 ± 40.4	108.9 ± 23.4
Mean Error: Palmar		104.8 ± 32.9	88.0 ± 29.8	83.9 ± 26.6
VPT (μm)^*^	***n***	9	11	7
D2		13.9 ± 9.4	10.5 ± 5.2	15.3 ± 12.8
D5		11.2 ± 5.5	8.1 ± 2.5	10.6 ± 4.7
**CPT** ( **μA) 5 Hz**	***n***	9	7	6
D2		28.9 ± 21.9	47.6 ± 17.2	38.3 ± 30.6
D5		48.6 ± 24.1	46.1 ± 37.3	32.7 ± 15.7
**CPT** ( **μA) 250 Hz**	***n***	7	8	7
D2		82.6 ± 39.0	86.8 ± 44.8	79.6 ± 46.7
D5		84.1 ± 23.9	60.0 ± 35.6	57.4 ± 15.3
**CPT** ( **μA) 2000 Hz**	***n***	5	6	3
D2		272.0 ± 71.2	250.0 ± 83.2	164.0 ± 126.1
D5		215.2 ± 15.3	165.3 ± 62.0	180.0 ± 10.6

* significant site main effect.

The effect sizes for group, site, and group-site interaction for the sensory measures are shown in Table 4.

**Table 4:** Effect size(d) for the neurophysiological and quantitative sensory tests

**Variable**	**Group**	**Site**	**Group-site**
SNAP amplitude	0.33	0.01	0.05
SNAP conduction velocity	0.007	0.38	0.06
SM	0.08	0.38	0.003
VPT	0.09	0.16	0.02
CPT-5 Hz	0.05	0.02	0.15
CPT-250 Hz	0.05	0.14	0.09

*SNAP* sensory nerve action potential; *SM* sensory mapping; *VPT* vibration perception threshold; *CPT* current perception threshold.

### Self-report disability, performance tests and neuromuscular recordings

The ANOVA results of the disability, strength, dexterity and neuromuscular tests are shown in Table 5. A significant group difference was found in the Hand and Finger Function scale (F(2,25) = 3.962, *p* = 0.033), where post hoc analysis revealed a significant difference between the healthy and hand RA groups ( *p* < 0.05), but not between the healthy and hand OA groups or between the hand OA and hand RA groups ( *p* > 0.05). The ANOVA for strength testing revealed no significant group differences (F(2,26) = 1.649, *p* = 2.13). A significant group difference was found for the dexterity task of the small (F(2,26) = 4.472, *p* = 0.022) and large (F(2,26) = 3.835, *p* = 0.036) objects. Post hoc analysis revealed the healthy group was significantly faster at completing the dexterity tasks with both small and large objects compared to the hand RA group ( *p* < 0.05), but not the hand OA group ( *p* > 0.05). No differences were found between the hand OA and hand RA groups for the dexterity task with small objects or large objects ( *p* > 0.05). The ANOVA revealed no significant group differences for CMAP conduction velocity (F(2,25) = 0.269, *p* = 0.767) or CMAP amplitude (F(2,25) = 1.721, *p* = 0.201).

**Table 5:** Mean values and SDs for the self-report disability questionnaire, performance tests and neuromuscular recordings for the healthy, hand OA and hand RA groups

**Variable**	**Healthy group** **n = 9**	**Hand OA group** **n = 11**	**Hand RA group** **n = 7**
**AIMS-2 hand/finger function**	NA	2.2 ± 1.9	3.5 ± 3.0
**Tripod pinch (kg)**	7.0 ± 1.4	6.0 ± 2.0	5.8 ± 3.1
**Grip strength (kg)**	27.7 ± 5.0	24.1 ± 6.8	21.1 ± 10.0
**Small dexterity time (s)**	38.9 ± 5.3	42.5 ± 5.5	52.8 ± 16.4*
**Large dexterity time (s)**	26.5 ± 4.1	31.4 ± 4.6	33.2 ± 6.9*
**CMAP1 Latency (ms)**	4.4 ± 0.8	4.1 ± 0.8	4.3 ± 0.6
**CMAP1Amplitude (mV)**	13.6 ± 5.3	12.6 ± 5.8	11.5 ± 4.7
**CMAP2 Latency (ms)**	8.5 ± 0.9	8.2 ± 0.9	8.5 ± 1.1
**CMAP2 Amplitude (mV)**	13.7 ± 5.8	14.5 ± 4.9	10.0 ± 4.2
**CMAP CV (m/s)**	57.0 ± 4.8	56.8 ± 5.3	59.5 ± 13.6

*significantly slower compared with the healthy group (*p* < 0.05).

In the group of women with hand RA, grip strength was associated with ulnar SNAP amplitude (*r* = 0.82, *p* < 0.05). No other significant relationships existed between SNAP amplitudes and self-report and performance tests.

## Discussion

The purpose of this study was to determine if women with OA or RA of the hand had sensory changes in the hand compared to women without hand problems using neurophysiological and sensory perception tests. Of all the tests performed, only the SNAP amplitudes in the median, ulnar and radial nerves were different in women with OA and RA of the hand compared to the healthy comparison group. The SNAP amplitudes represent the number of active sensory nerve fibers. Therefore, lower amplitude will correspond with decreased number of active fibers [36]. An association between ulnar SNAP amplitude and grip strength was also observed. However, it appears that sensory perception has not changed in these women with hand arthritis despite a loss in sensory units (as indicated by the reduced SNAP amplitudes). Whether sensory loss is related to disuse due to joint pain or is an inherent part of the disease remains unknown. However, the consequence of sensory unit loss does appear to affect hand activity for women with RA of the hand, at least as indicated by the significant correlation with grip strength. The differences found between the hand RA and healthy groups but not the hand OA and healthy groups may be related to the longer disease duration and higher disability self-reported by the hand RA group. This finding warrants further investigation in women having a longer duration of hand OA and/or greater hand disability to determine if these observations apply to women with hand OA as well.

Even though there were differences in SNAP amplitudes between groups, there were no significant differences in the SNAP conduction velocities. The observed pattern of SNAP amplitude reduction with conserved conduction velocity is typically associated with axonal loss lesions [36,49], and differs from the pattern seen in demyelinating neuropathies. This is consistent with our screening to rule out compression neuropathy as an exclusion criterion. While there may be fewer total nerve fibers firing, the remaining intact fibers have normal nerve conduction [36,49]. The findings from this study suggest that women with hand OA and hand RA may have axonal loss of sensory fibers in the median, ulnar and radial nerves.

The sensory outcome measures used in this study record mainly the activity of larger diameter afferent fibers of the wrist and hand [50]. However, the SNAP amplitude comprises action potentials of fibers of all sizes. The lack of group differences for the majority of tests would suggest that, in women with hand OA and hand RA, these larger fibers may not be significantly impaired [16,17,49]. The significant decrease in SNAP amplitudes in the hand OA group and hand RA group may be a result of the loss of small, slower conducting sensory nerve fiber axons such as C-fibers stimulated by temperature and pain [36]. If the C-fibers are affected in hand OA and hand RA, the CPT at 5 Hz should have been higher for our participants with hand arthritis compared to our healthy participants, because this test is intended to evoke sensory responses from unmyelinated C-fibers [43]. However the CPT test required active subject participation over a period of 30 minutes or longer. Lack of attention or motivation may result in inconsistent results [51]. Indeed, results were available for only a subset of our participants due to inconsistent readings, especially at 2000 Hz. Kang et al.[43] have reported difficulties in using CPT to evaluate carpal tunnel syndrome and suggest that nerve conduction testing is a better measure for this purpose. A review by the American Association of Electrodiagnostic Medicine (AAEM) concluded that the information regarding the usefulness of CPT for sensory testing is insufficient [51]. To address these concerns we incorporated three other methods to measure sensory impairment in the current study.

The significant site differences found on SM and VPT testing are not surprising as the palmar hand has more sensory receptors than the dorsal hand, and digit has more sensory receptors than digit [52,53]. The SNAP latencies for all groups were found to be within the normal range, as the median nerve SNAP relative to the ulnar nerve SNAP did not exceed 0.5 ms, the radial nerve SNAP did not exceed 2.9 ms, and the radial nerve latency did not exceed the median nerve latency [36]. Although there were no differences in SNAP latencies or conduction velocities between the groups, a significant site effect was observed. The radial SNAP conduction velocity was greater than the median and ulnar nerves. This finding was unexpected, however the device used to elicit a response for the radial nerve SNAP was different than the device used for the median and ulnar SNAP recordings (Figure 1a,b). The pressure applied using the stimulating probe on the radial nerve may provide more direct contact on the nerve. The absolute mean values for median and ulnar nerve conduction velocities were low at 42.7 and 43 m/s, respectively, when compared to data published by Tong et al. (2004) where the conduction velocity was 49.23 ± 6.74 m/s for median sensory response and 50.82 ± 6.03 m/s for the ulnar sensory response [54]. However, the average age of the 440 participants in the previous study was 38.4 ± 9.7 years, and age-related decreases in median and ulnar sensory conduction velocities were estimated to be 0.13 to 0.21 m/s per year [54]. Effectively, this is a decrease in conduction velocities of 10 m/s or less even into the ninth decade. On average, the women in our study were approximately 20 years older (healthy group: 56.67 ± 5.50 yrs; hand OA group: 57.00 ± 5.33 yrs; hand RA group: 60.43 ± 5.00 yrs). If we correct for this age difference then the conduction velocity would be around 46.9 m/s for the median nerve and 47.2 m/s for the ulnar nerve. With the conduction velocities adjusted for age, our values are comparable to previously published values [54].

Although there was a trend towards lower hand strength in the hand OA and hand RA groups, no significant differences were found between groups. Using maximal grip strength as an example, the mean grip strength in the healthy group (27.6 kg) was consistent with published normative data (26 kg) [55]. Mean grip strength in our group of women with hand OA (24.1 kg) was greater than that reported previously for women with hand OA (21.5 kg) [56]. Similarly, grip strength in our women with hand RA (21.6 kg) was greater than that reported previously (12.2 kg) [19]. Despite the non-significant differences in the pinch and grip strength, the hand RA group performed significantly more slowly in the timed dexterity tasks for both the small and large objects when compared to the healthy group. We attribute the relatively strong grip strength measures in our sample to be partially related to our recruitment of community-based volunteers who would represent the mild end of the spectrum of arthritis. Since we were interested in early manifestations of sensory problems in hand arthritis, this finding confirms that we were able to target our recruitment appropriately.

It appears that hand strength (although not significantly different between groups) is related to the level of self-reported hand disability. The self-reported hand disability scores in our participants with hand OA (2.23 ± 1.90) were lower than reported in a previous study for women with hand OA, a group with moderate severity based on Hand and Finger Function scale scores (3.08 ± 2.06) [57]. The self-reported disability score in our hand RA group (3.5 ± 2.95) was similar to that reported in a previous study of women with hand RA participants (3.16 ± 2.58) [44]. All our participants with hand arthritis scored <5.0 points on the Hand and Finger Function scale (where 10 is severe disability), suggesting moderate to low levels of disability.

Even though our participants with OA and RA of the hand had low disability on average, the SNAP test was sensitive enough to measure differences between those groups with arthritis of the hand and the healthy comparison group. The results of this study suggest that sensory changes occur before motor changes, a common pattern for neuropathic conditions. The CMAP data revealed no differences between our three groups indicating the number of functioning motor units in the thenar muscle is similar in all the groups. All groups were within the normal limits for latency (normal upper limit of 4.4 ms), amplitude (normal lower limit of 4 mV), and conduction velocity (exceeds 49 m/s) [36]. These findings are consistent with the strength results which indicated no differences between groups.

The SNAP amplitude was the only sensory measure significantly different among groups, and, in the ulnar nerve, was positively related to the grip strength in the hand RA group. This relationship suggests that the sensory fibers of the ulnar nerve, providing sensory innervation to the fifth digit and medial palmar region of the hand, are associated with increased grip strength. So if there is deterioration in sensory acuity then that could manifest as a decline in motor function—hence slower dexterity in the hand RA participants. Based on our observations in women with good grip and pinch strength, dexterity testing, a simultaneous assessment of motor and sensory function, was a better indicator of early sensory changes than assessment of sensory perception threshold. Thus, dexterity testing may be a viable option for detecting sensory problems during physical assessment.

The primary limitations of this study are the small sample size and lack of range in severity of hand OA and RA. Our sensory perception findings were inconclusive and demonstrated more variability than the nerve conduction findings. Larger clinical studies are needed to determine whether observed group differences in sensory perception tests are significant. Larger longitudinal investigations using nerve conduction studies and measuring motor ability would allow for stratification by severity, and provide insight into the temporal course of sensorimotor changes. Another important consideration for future work is to take into account palmar digital skin thickness using equipment such as a sonography machine and/or skin fold calipers. Swelling or nodes can significantly increase the size of the finger which may reduce SNAP amplitudes as has been observed in heavier people, people with larger finger circumference and people with thicker skin [58]. In the current study, a physical therapist performed a clinical examination of the arthritic hands, and qualitatively reported joint involvement based on nodes and swelling (Table 1). We do not believe that swelling or nodes had a significant impact in this pilot study, as only two participants had nodes on digit 4, and no swelling was noted on digit 4. For the radial SNAP recording, one of the participants with hand RA had swelling at the MCP joint, and two participants with hand RA had nodes at the MCP joint, but the recording electrode was positioned below the MCP at the ‘snuff box’. However, for future investigations involving SNAP analyses, quantitative measures of the size and skin thickness in the recording finger is recommended. Evaluation of sensory changes in hand OA and RA using outcomes such as pain, temperature and nerve conduction tests using needle electrodes to measure the small, late components of SNAPS (smaller nerve fibres) is needed to confirm and extend these findings.

## Conclusions

The goal of this study was to determine if women with OA or RA of the hand had sensory deficits, and this was confirmed with neurophysiological tests. The results indicate the importance of incorporating neurophysiological investigations when studying sensory and motor factors that may contribute to disability and limitation in activities in persons with hand OA and RA. Results of this study may have significant implications both for the assessment of sensory deficits in hand OA and RA and for our understanding of the pathophysiology, onset, progression and rehabilitation of these diseases.

## Abbreviations

AIMS-2: Arthritis Impact Measurement Scales 2; ANOVA: Analysis of variance; CMAP: Compound muscle action potential; CPT: Current perception threshold; OA: Osteoarthritis; RA: Rheumatoid arthritis; SM: Sensory mapping; SNAP: Sensory nerve action potential; VPT: Vibratory perception threshold.

## Competing interests

The authors declare that they have no competing interests.

## Authors’ contributions

KMC, AM, JL, NJM carried out the recruitment and testing of participants, acquisition of data, analysis and interpretation of data. KMC wrote the manuscript. KMC, VG, JCM, NJM conceptualized the research question and study design, and provided guidance in terms of data acquisition, analysis and interpretation. All authors were active in the editing process of the manuscript. All authors read and approved the final manuscript.
